# Identification of BCL11B as a regulator of adipogenesis

**DOI:** 10.1038/srep32750

**Published:** 2016-09-02

**Authors:** Jun Inoue, Yusuke Ihara, Daisuke Tsukamoto, Keisuke Yasumoto, Tsutomu Hashidume, Kenya Kamimura, Yuji Nakai, Shigeki Hirano, Makoto Shimizu, Ryo Kominami, Ryuichiro Sato

**Affiliations:** 1 Department of Applied Biological Chemistry, Graduate School of Agricultural and Life Sciences, The University of Tokyo, Tokyo, Japan; 2 Institute of Gerontology, The University of Tokyo, Tokyo, Japan; 3 Division of Gastroenterology and Hepatology, Graduate School of Medical and Dental Sciences, Niigata University, Niigata, Japan; 4 Institute for Food Sciences, Hirosaki University, Aomori, Japan; 5 Department of Medical Technology, School of Health Sciences, Faculty of Medicine, Niigata University, Niigata, Japan; 6 Division of Molecular Biology, Department of Molecular Genetics, Graduate School of Medical and Dental Sciences, Niigata University, Niigata, Japan

## Abstract

The differentiation of preadipocytes into adipocytes is controlled by several transcription factors, including peroxisome proliferator-activated receptor γ (PPARγ) and CCAAT/enhancer-binding protein α (C/EBPα), which are known as master regulators of adipogenesis. BCL11B is a zinc finger-type transcription factor that regulates the development of the skin and central nervous and immune systems. Here, we found that BCL11B was expressed in the white adipose tissue (WAT), particularly the subcutaneous WAT and that *BCL11B*^−/−^ mice had a reduced amount of subcutaneous WAT. During adipogenesis, BCL11B expression transiently increased in 3T3-L1 preadipocytes and mouse embryonic fibroblasts (MEFs). The ability for adipogenesis was reduced in *BCL11B* knockdown 3T3-L1 cells and *BCL11B*^−/−^ MEFs, whereas the ability for osteoblastogenesis was unaffected in *BCL11B*^−/−^ MEFs. Luciferase reporter gene assays revealed that BCL11B stimulated C/EBPβ activity. Furthermore, the expression of downstream genes of the Wnt/β-catenin signaling pathway was not suppressed in *BCL11B*^−/−^ MEFs during adipogenesis. Thus, this study identifies BCL11B as a novel regulator of adipogenesis, which works, at least in part, by stimulating C/EBPβ activity and suppressing the Wnt/β-catenin signaling pathway.

Obesity is increasing worldwide, resulting in increased incidence of metabolic complications^1^. Obesity is an energy balance disorder that results in excess energy accumulating as fat in the white adipose tissue (WAT). Adipocytes play a role in storing fat in the WAT^2^ and differentiate from mesenchymal stem cells (MSCs) via a process known as adipogenesis. Therefore, an accurate understanding of the molecular mechanisms involved in adipogenesis is required to develop methods that target obesity. In-depth molecular investigations of the differentiation of preadipocytes into adipocytes have shown that several transcription factors are involved in this process. The expression of CCAAT/enhancer-binding protein β (C/EBPβ) and C/EBPδ increases during the early stages of adipogenesis. These proteins induce the expression of peroxisome proliferator-activated receptor γ (PPARγ) and C/EBPα, which regulate the expression of genes that control cell differentiation into adipocytes^3^. It is also known that other transcription factors, such as Krupel-like factors (KLFs) and Wnt/β-catenin signaling, regulate adipogenesis^3^,^4^. However, the exact process is not yet entirely clear.

Binding of the Wnt ligand to its receptors, frizzled and low-density lipoprotein receptor-related protein 5/6 (LRP5/6) complexes, stimulates the Wnt/β-catenin signaling pathway and consequently activates T-cell factor (Tcf)-β-catenin-mediated transcription^5^. This pathway plays a pivotal role in the commitment and differentiation of MSCs^4^. The activation of Wnt/β-catenin signaling stimulates differentiation of MSCs into osteoblasts and myoblasts, whereas its suppression promotes their differentiation into adipocytes^4^. Wnt10b is expressed in 3T3-L1 preadipocytes and maintains these cells in an undifferentiated state through the suppression of PPARγ and C/EBPα expression^6^ and is thus considered a candidate for an anti-adipogenic Wnt ligand. In contrast, expression of the Wnt inhibitor Dickkopf-1 is induced during the early stages of adipogenesis, and this induction is required for the proper progression of adipogenesis^7^,^8^.

BCL11B, which is also known as COUP-TF-interacting protein 2 (CTIP2) and radiation induced tumor suppressor gene (Rit1), is a transcription factor that has six C_2_H_2_-type zinc finger domains and was discovered by two independent groups using different approaches^9^,^10^. BCL11A/CTIP1 and BCL11B/CTIP2 were identified as binding partners with the nuclear receptors COUP-TFs^9^. There are three types of COUP-TFs (COUP-TFI, -TFII, and -TFIII), which commonly function as transcriptional repressors. Thus, it is considered that BCL11A/CTIP1 and BCL11B/CTIP2 function as co-repressors of COUP-TFs^9^. In addition, BCL11B/CTIP2 can directly bind to the GC-rich DNA sequence of the promoter region of certain genes, suppressing their promoter activities through the recruitment of the histone deacetylase sirtuin 1 (SIRT1) and the nucleosome remodeling and deacetylation (NuRD) complex^11^,^12^,^13^. We have previously reported that *BCL11B/Rit1* is found as a tumor suppressor gene in γ-ray-induced mouse thymic lymphomas^10^. BCL11B/Rit1 acts as a haploinsufficient tumor suppressor in mouse thymic lymphomas^14^ and human T-cell acute lymphoblastic leukemia^15^,^16^. BCL11B is expressed in various tissues, such as the central nervous system, thymus, and epidermis^17^,^18^, and a loss in the *BCL11B* gene results in abnormal development of the T cells^19^,^20^,^21^. corticospinal motor neurons^22^, skin^23^, and teeth^24^.

We accidentally found that *BCL11B*-deficient mice have abnormal subcutaneous WAT, and subsequent experiments showed that BCL11B expression is transiently induced in the early stages of adipogenesis. We also found that the Wnt/β-catenin signaling pathway is attenuated in *BCL11B*^+/+^ mouse embryonic fibroblasts (MEFs) during adipogenesis but not in MEFs from *BCL11B*^−/−^ embryos and that unexpectedly this loss of *BCL11B* gene does not affect osteoblastogenesis in MEFs.

## Results

### BCL11B is expressed in the white adipose tissue (WAT), and *BCL11B* ^−/−^ mice have abnormal WAT

**Figure 1:**
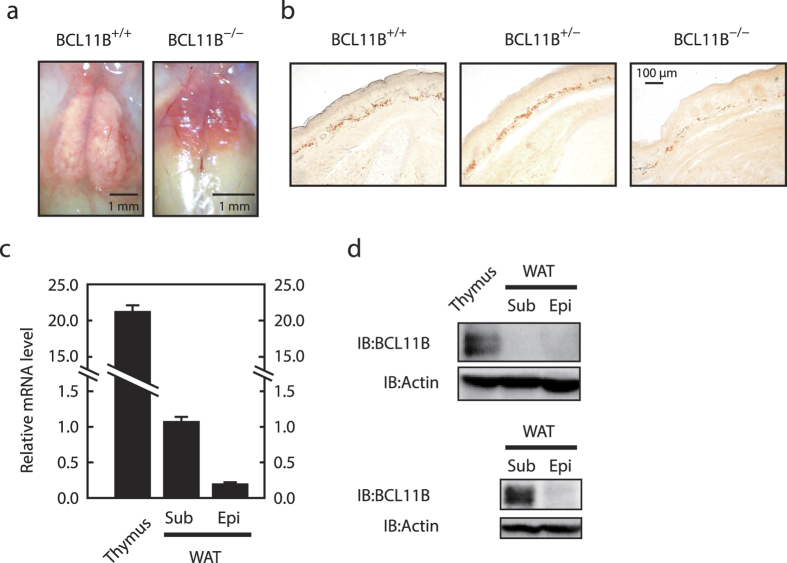
Morphology and histology of the white adipose tissue (WAT) from *BCL11B*^−/−^, *BCL11B*^+/−,^ and *BCL11B*^+/+^ mice, and the expression of BCL11B in the WAT of adult mice. (**a**) Morphology of the interscapular WAT from neonatal *BCL11B*^+/+^ and *BCL11B*^−/−^ mice. (**b**) Sections from *BCL11B*^+/+^. *BCL11B*^+/−^, and *BCL11B*^−/−^mice at E19.5 stained with Sudan III, as described under “Methods.” (**c**) Relative mRNA levels of *BCL11B* in the thymus, subcutaneous (sub) WAT, and epididymal (epi) WAT of adult mice (8-week-old males), assessed using real-time PCR analysis with normalization to *18S* rRNA. The mRNA levels of the subcutaneous WAT are represented as 1. All data are expressed as means ± S.E. (n = 3). (**d**) BCL11B protein content of whole cell extracts from the thymus, subcutaneous (sub) WAT, and epididymal (epi) WAT of adult mice (8-week-old males), assessed using SDS−PAGE and immunoblotting (IB) with anti-BCL11B or anti-β-actin antibodies. Shown here are representative results of at least three replicate experiments.

All of the *BCL11B*^−/−^ mice died within 24 h of birth for unknown reasons. Within 24 h of birth, the live *BCL11B*^−/−^ mice possessed shrunken interscapular WAT (Fig. 1a), which we attributed to the lower nutritional status of these mice, which are unable to ingest breast milk. Therefore, to circumvent this problem, we observed the subcutaneous WAT at E19.5. We found that both *BCL11B*^+/+^ and *BCL11B*^+/−^ mice possessed a substantial subcutaneous WAT that stained red with Sudan III, whereas *BCL11B*^−/−^ mice had a very low amount of this (Fig. 1b). It should be noted that the epidydimal WAT does not develop sufficiently for observation in embryos at E19.5 and newborn mice. Although *BCL11B* has previously been reported as being expressed in various tissues, including the thymus, skin, and central nervous system^17^,^18^, it has not previously been clear whether it is also expressed in the WAT. Therefore, we examined the expression of *BCL11B* in the WAT of adult mice.

Quantitative real-time PCR analyses revealed that *BCL11B* is expressed in the subcutaneous WAT, although the expression level is relatively low compared with that of the thymus (Fig. 1c). In addition, the mRNA expression of *BCL11B* in the epididymal WAT was one-tenth that in the subcutaneous WAT (Fig. 1c). We observed a similar expression profile using immunoblotting, with the expression of the BCL11B protein in the subcutaneous and epididymal WAT not being detected when the samples were analyzed on the same membrane as the thymus sample due to the relatively high expression in the latter (Fig. 1d upper panel). However, when we excluded the thymus sample from the analysis, the BCL11B protein was detected in the subcutaneous WAT, but not the epididymal WAT (Fig. 1d lower panel). These results indicate that BCL11B is expressed at a higher level in the subcutaneous WAT than that in the epididymal WAT in adult mice.

### BCL11B expression transiently increases during adipocyte differentiation, and the knockdown of *BCL11B* results in the attenuation of adipocyte differentiation in 3T3-L1 preadipocytes

**Figure 2:**
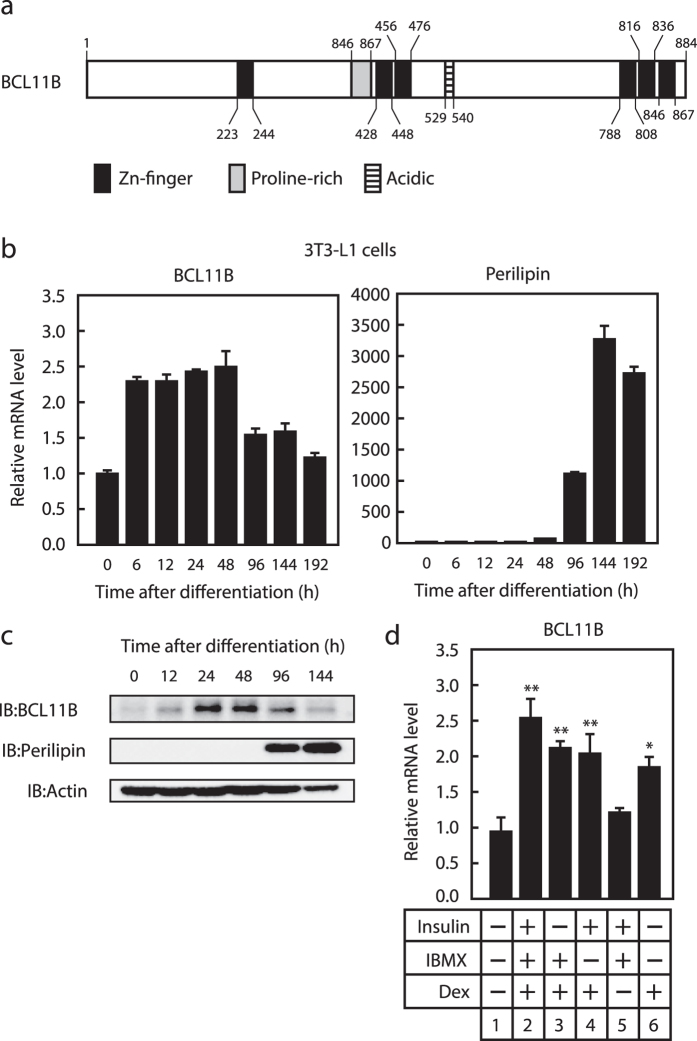
Transient expression of BCL11B during adipogenesis. (**a**) Diagrammatic representation of the BCL11B protein. (**b,c**) Total RNA and protein were isolated from 3T3-L1 cells at the indicated times after treating them for adipocyte differentiation, as described under “Methods.” (**b**) Relative mRNA levels of *BCL11B* and *perilipin*, assessed using real-time PCR analysis with normalization to *18S* rRNA. mRNA levels in cells that were not treated for adipocyte differentiation are represented as 1. (**c**) BCL11B protein content of whole cell extracts assessed using SDS−PAGE and immunoblotting (IB) with anti-BCL11B, anti-Perilipin, or anti-β-actin antibodies. Shown here are representative results of at least three replicate experiments. (**d**) Relative mRNA levels of *BCL11B* in 3T3-L1 cells cultured in individual or multiple components of the induction cocktail (insulin, IBMX, and dexamethasone [DEX]) for 48 h. Total RNA was isolated, and real-time PCR analysis of *BCL11B* was performed as described in (**b**). All data are expressed as means ± S.E. (n = 3). *P < 0.05; **P < 0.01.

The three consecutive zinc-finger domains in the C-terminal region of BCL11B are a distinctive feature of this protein (Fig. 2a). The KLF family contains three similar consecutive zinc-finger domains and several recent reports have indicated that these are involved in adipogenesis, with KLF4, KLF5, KLF6, and KLF15 stimulating adipogenesis and KLF2 and KLF3 suppressing it^25^. Thus, we examined the effect of BCL11B on adipogenesis using 3T3-L1 preadipocytes. We first investigated the expression of *BCL11B* during adipogenesis. We differentiated the 3T3-L1 cells into adipocytes by treating them with an induction cocktail that included insulin, dexamethasone, and IBMX, and then collected the total mRNA and proteins at specific time points (Fig. 2b,c). We found that the gene expression of *BCL11B* increased approximately 2.5-fold at 6 h. This induction then continued until 48 h after differentiation (Fig. 2b), but was attenuated at 96 h, and had disappeared by 192 h after differentiation. Levels of the BCL11B protein also transiently increased during adipogenesis, peaking at 24 and 48 h after differentiation (Fig. 2c). It should be noted that expression of the adipocyte marker perilipin also increased after differentiation, suggesting that the cells had correctly differentiated into adipocytes (Fig. 2b,c).

We then examined the stimuli that are responsible for inducing *BCL11B* expression during adipogenesis. As shown in Fig. 2d, when insulin or IBMX were removed from the hormonal cocktail, the mRNA level of *BCL11B* still increased (lanes 3 and 4), whereas when dexamethasone was removed, it did not (lane 5). In addition, treatment with dexamethasone alone increased *BCL11B* gene expression (lane 6). These results indicate that *BCL11B* expression is transiently increased during adipogenesis in 3T3-L1 preadipocytes and that dexamethasone is responsible for this induction.

**Figure 3:**
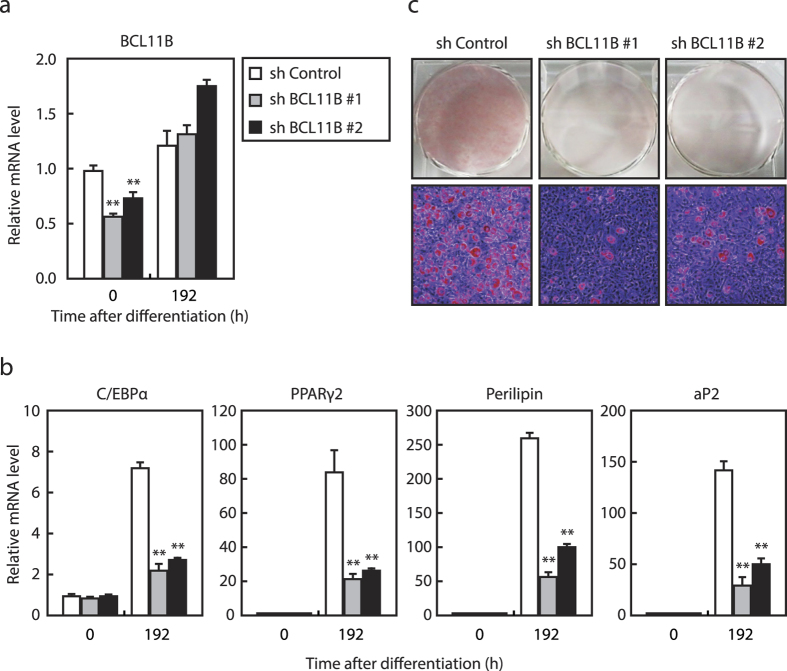
Effect of knockdown of *BCL11B* in 3T3-L1 cells on adipogenesis. 3T3-L1 cells were infected with a lentivirus expressing either control shRNA (shControl) or shRNA for *BCL11B* (shBCL11B#1 or shBCL11B#2). The cells were differentiated into adipocytes as described under “Methods.” (**a**) Relative mRNA levels of *BCL11B* in the control and knockdown cells at the indicated times after treatment for adipocyte differentiation, assessed using real-time PCR. (**b**) Relative mRNA levels of the adipocyte marker genes *C/EBPα, PPARγ2, perilipin*, and *aP2* in the control and knockdown cells at the indicated times after treatment for adipocyte differentiation, assessed using real-time PCR. The mRNA levels of 3T3-L1 cells in the control shRNA group that had not been treated for adipocyte differentiation are represented as 1. All data are expressed as means ± S.E. (n = 3). **P < 0.01. (**c**) Control and knockdown cells stained with Oil Red O at 192 h (day 8) after differentiation.

Next, we examined the influence of *BCL11B* knockdown on adipogenesis by infecting 3T3-L1 cells with a lentivirus expressing shBCL11B or shRNA as a control. We found that the mRNA level of *BCL11B* was reduced by 50% by shBCL11B#1 and 30% by shBCL11B#2 at 0 h after differentiation; however, these knockdown effects disappeared at 192 h after differentiation (Fig. 3a). By contrast, the mRNA levels of the adipocyte marker genes *C/EBPα, PPARγ2, perilipin*, and *aP2* were induced at 192 h after differentiation, but these inductions were attenuated by the knockdown of *BCL11B* (Fig. 3b). Consistent with these results, we also found that the formation of intracellular lipid droplets by adipogenesis as assessed by Oil Red O staining was reduced in *BCL11B*-knockdown cells (Fig. 3c), suggesting that the knockdown of *BCL11B* attenuates adipogenesis in 3T3-L1 preadipocytes.

### Adipogenesis is reduced in MEFs from *BCL11B* ^−/−^ embryos

**Figure 4:**
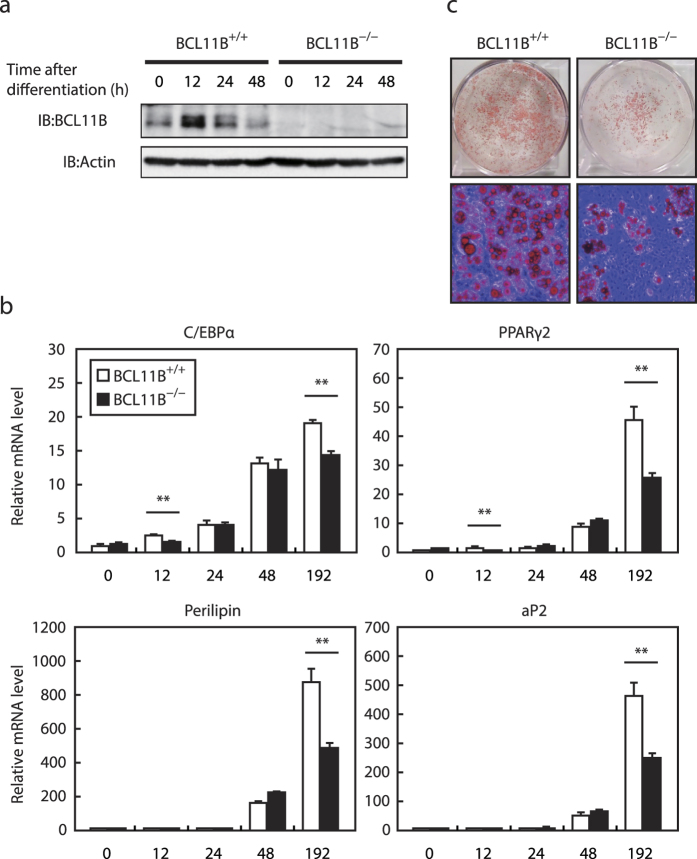
Adipocyte differentiation in *BCL11B*^−/−^ mouse embryonic fibroblasts (MEFs). Total RNA and protein were isolated from MEFs prepared from *BCL11B*^+/+^ and *BCL11B*^−/−^ embryos at the indicated times after treatment for adipocyte differentiation, as described under “Methods.” (**a**) BCL11B protein content of whole cell extracts assessed using SDS−PAGE and immunoblotting (IB) with anti-BCL11B or anti-β-actin antibodies. Shown here are representative results of at least three replicate experiments. (**b**) Relative mRNA levels of the adipocyte marker genes *C/EBPα, PPARγ2, perilipin*, and *aP2*, assessed using real-time PCR with normalization to *18S* rRNA. The mRNA levels of *BCL11B*^+/+^ MEFs that had not been treated for adipocyte differentiation are represented as 1. All data are expressed as means ± S.E. (n = 3). *P < 0.05; **P < 0.01. (**c**) Control and knockdown cells stained with Oil Red O at 192 h (day 8) after differentiation.

To confirm the importance of *BCL11B* in adipogenesis, we used primary MEFs prepared from *BCL11B*^−/−^ embryos at E12.5. The expression of the BCL11B protein transiently increased during adipogenesis in *BCL11B*^+/+^ MEFs (Fig. 4a). It is noteworthy that it also transiently increased in 3T3-L1 cells, although the peak time was different (24–48 h in 3T3-L1 cells vs. 12 h in *BCL11B*^+/+^ MEFs; Figs 2c and 4a). By contrast, the BCL11B protein was not present at a detectable level in *BCL11B*^−/−^ MEFs (Fig. 4a). Similarly, while the mRNA levels of the adipocyte differentiation markers *C/EBPα, PPARγ2, perilipin*, and *aP2,* were induced during adipogenesis in *BCL11B*^+/+^ MEFs, these inductions were attenuated at 192 h after differentiation in *BCL11B*^−/−^ MEFs (Fig. 4b). The formation of intracellular lipid droplets as a result of adipogenesis was also lower in *BCL11B*^−/−^ MEFs than in *BCL11B*^+/+^ MEFs (Fig. 4c). These results indicate that the expression of BCL11B transiently increases during adipogenesis, and that BCL11B stimulates adipogenesis in MEFs.

### Enforced expression of BCL11B does not stimulate adipogenesis

**Figure 5:**
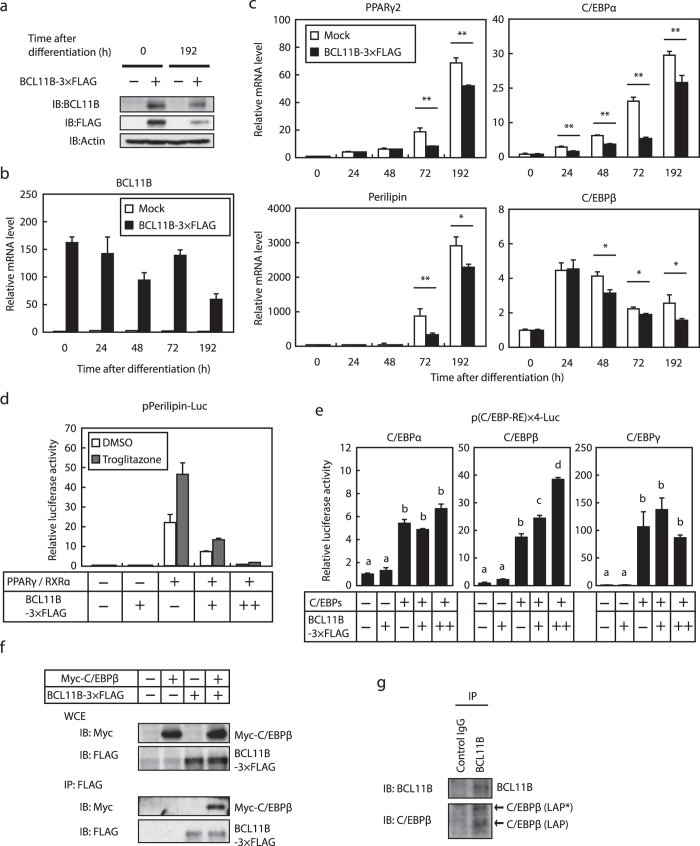
Effect of the enforced expression of BCL11B on adipocyte differentiation and the activity of adipogenic transcription factors. (**a–c**) 3T3-L1 cells were infected with a retrovirus that expressed BCL11B-3 × FLAG or was empty (Mock). The cells were then differentiated into adipocytes, as described under “Methods.” (**a**) BCL11B protein content of whole cell extracts at the indicated times after treatment for adipocyte differentiation, assessed using SDS−PAGE and immunoblotting (IB) with anti-BCL11B, anti-FLAG, or anti-β-actin antibodies. Shown here are representative results of at least three replicate experiments. (**b,c**) Relative mRNA levels of *BCL11B* (**b**) and the adipocyte marker genes *PPARγ2, perilipin, C/EBPα, and C/EBPβ* at the indicated times after treatment for adipocyte differentiation, assessed using real-time PCR. All data are expressed as means ± S.E. (n = 3). **P < 0.01. (**d,e**) Relative luciferase activity of 3T3-L1 cells transfected with 160 ng of the indicated reporter constructs; 0, 40, or 160 ng of pBCL11B-3 × FLAG (represented as −,+ , and + +, respectively); 160 ng of pCMV-β-gal; and either 160 ng of pPPARγ and pRXRα (**d**) or 160 ng of the indicated C/EBPs expressing plasmid (**e**). The cells were either cultured with medium A for 24 h and then treated with or without 10 μM troglitazone for 24 h (**d**) or cultured with medium A for 48 h. Luciferase assays were performed as described under “Methods.” All data are expressed as means ± S.E. (n = 3). Bars with different letters are significantly different (*P* < 0.05). (**f**) HEK293 cells were transfected with expression plasmids for Myc-C/EBPβ and BCL11B-3 × FLAG, and the whole cell extract (WCE) was subjected to immunoprecipitation (IP) with anti-FLAG antibodies. Aliquots of whole cell extracts and pellets of immunoprecipitates were subjected to SDS-PAGE and immunoblotting (IB) with anti-c-Myc and anti-FLAG antibodies. (**g**) 3T3-L1 cells were differentiated into adipocytes for 24 h, and then the cells were lysed and immunoprecipitated with anti-BCL11B antibodies and immunoblotted with antibodies with anti-BCL11B and anti-C/EBP antibodies.

We next investigated the effect of the enforced constitutive expression of BCL11B on adipogenesis by infecting 3T3-L1 cells with a retrovirus expressing BCL11B-3 × Flag, and then differentiating these into adipocytes. The protein and mRNA expressions of exogenous BCL11B-3 × Flag were detected at the indicated times after differentiation (Fig. 5a,b). It should be noted that the mRNA levels of exogenous BCL11B was 150-fold that of endogenous BCL11B. Unexpectedly, the mRNA levels of the adipocyte differentiation marker genes *PPARγ2, perilipin, C/EBPα*, and *C/EBPβ* were not induced by the enforced expression of BCL11B, but rather significantly decreased (Fig. 5c). To determine the molecular mechanism by which the enforced expression of BCL11B suppresses the expression of adipocyte marker genes during adipogenesis, we examined the effect of BCL11B on the activities of the adipogenic transcription factors PPARγ, C/EBPα, C/EBPβ, and C/EBPδ. Luciferase reporter gene assays revealed that BCL11B suppressed PPARγ activity in the presence or absence of its ligand, troglitazone (Fig. 5d). By contrast, BCL11B stimulated C/EBPβ activity, but had no effect on the activities of C/EBPα and C/EBPδ (Fig. 5e). These results indicate that the enforced expression of BCL11B positively and negatively regulates various adipogenic transcription factors and so does not stimulate adipogenesis.

Given that BCL11B expression transiently increased during the early stages of adipogenesis, it is probable that endogenous BCL11B affects C/EBPβ activity. Thus, we next examined whether BCL11B can associate with C/EBPβ. When BCL11B-3 × FLAG and Myc-C/EBPβ were expressed in HEK293 cells, BCL11B was co-immunoprecipitated with C/EBPβ (Fig. 5f). In addition, endogenous BCL11B was co-immunoprecipitated with C/EBPβ (Fig. 5g), indicating that BCL11B and C/EBPβ can form a complex.

### The Wnt/β-catenin signaling pathway is not suppressed properly during adipocyte differentiation in *BCL11B* ^−/−^ MEFs

**Figure 6:**
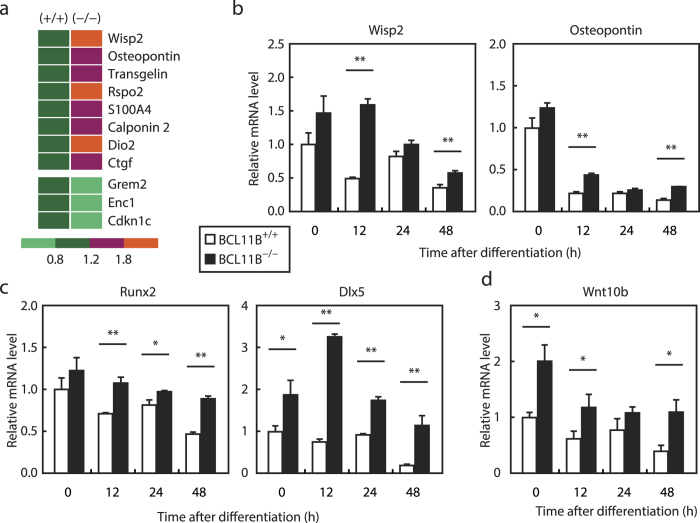
Effect of *BCL11B* deficiency on the Wnt/β-catenin signaling pathway during adipocyte differentiation. (**a**) A heat map showing the expression levels of mRNA regulated by Wnt/β-catenin signaling at 12 h after adipocyte differentiation in *BCL11B*^+/+^ and *BCL11B*^−/−^ mouse embryonic fibroblasts (MEFs). These expression levels represent normalized values obtained from the Affymetrix arrays. (**b–d**) Total RNA was isolated from MEFs prepared from *BCL11B*^+/+^ and *BCL11B*^−/−^ embryos at the indicated times after treatment for adipocyte differentiation, as described under “Methods.” Relative mRNA levels were then measured using real-time PCR analysis with normalization to *18S* rRNA. (**b**) Relative mRNA levels of the Wnt/β-catenin signaling downstream genes *Wisp2* and *Osteopontin*. (**c**) Relative mRNA levels of the osteoblast marker genes *Runx2* and *Dlx5*. (**d**) Relative mRNA levels of *Wnt10b.* The mRNA levels of *BCL11B*^+/+^ MEFs that were not treated for adipocyte differentiation are represented as 1. All data are expressed as means ± S.E. (n = 3). *P < 0.05; **P < 0.01.

To gain further insight into the mechanism by which BCL11B regulates adipogenesis, we performed DNA microarray analysis using *BCL11B*^+/+^ and *BCL11B*^−/−^ MEFs at 12 h after differentiation, which is the peak time of BCL11B expression (Fig. 4a). This showed that a Wnt/β-catenin signaling pathway appeared to be activated at 12 h after differentiation in *BCL11B*^−/−^ MEFs compared with *BCL11B*^+/+^ MEFs (*P* < 0.01). The top 100 genes as evaluated by weighted average difference (WAD) included 11 downstream genes of the Wnt/β-catenin signaling pathway (Fig. 6a). Eight of these genes, including *Wisp2* and *Osteopontin*, are reportedly stimulated by Wnt/β-catenin signaling, and exhibited increased expression (Fig. 6a upper panel), whereas the remaining three genes (*Grem2, Enc1*, and *Cdkn1c*) are reportedly suppressed by Wnt/β-catenin signaling and exhibited decreased expression (Fig. 6a lower panel) at 12 h after differentiation in *BCL11B*^−/−^ MEFs.

To confirm the microarray result, we performed real-time PCR analyses of *BCL11B*^+/+^ and *BCL11B*^−/−^ MEFs samples collected at set time points after differentiation (Fig. 6b). Consistent with the microarray results, this showed that the mRNA levels of *Wisp2* and *Osteopontin* significantly increased at 12 h after differentiation in *BCL11B*^−/−^ MEFs. Furthermore, and importantly, the mRNA levels of these genes decreased during adipogenesis in *BCL11B*^+/+^ MEFs, but these decreases were attenuated in *BCL11B*^−/−^ MEFs (Fig. 6b), implying that *BCL11B* suppresses the Wnt/β-catenin signaling pathway during adipogenesis.

Since the activation of Wnt/β-catenin signaling has previously been shown to promote the differentiation of mesenchymal precursor cells into osteoblasts while suppressing their commitment to adipocyte^4^, we analyzed the expression of the osteoblast marker genes *Runx2* and *Dlx5*. Whereas the expression of these genes decreased in *BCL11B*^+/+^ MEFs during adipogenesis, *BCL11B*^−/−^ MEFs had significantly larger levels of expression for these genes (Fig. 6c). In addition, the expression of *Wnt10b*, an endogenous Wnt isoform in preadipocytes, significantly increased in *BCL11B*^−/−^ MEFs, and this increase continued during adipogenesis (Fig. 6d).

### A loss in the *BCL11B* gene does not affect osteoblastogenesis in MEFs

**Figure 7:**
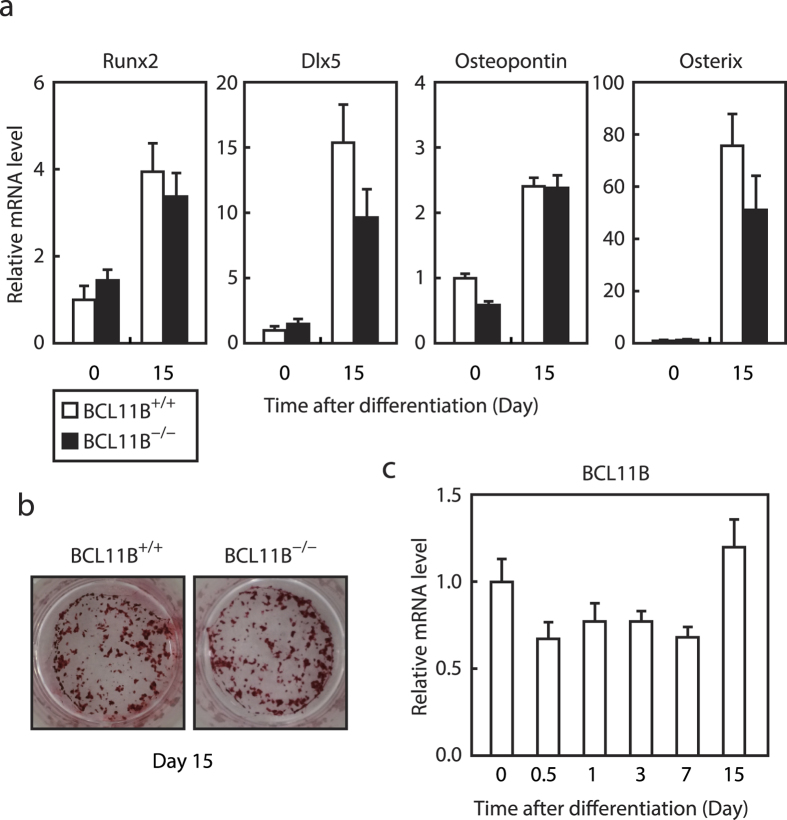
Effect of *BCL11B* deficiency on osteoblast differentiation. *BCL11B*^+/+^ and *BCL11B*^−/−^ mouse embryonic fibroblasts (MEFs) were differentiated into osteoblasts, as described under “Methods.” (**a,c**) mRNA levels of the osteoblast marker genes *Runx2, Dlx5, Osteopontin*, and *Osterix* (**a**), and *BCL11B* (**c**), analyzed at the indicated times after treatment for osteoblast differentiation using real-time PCR. The mRNA levels of *BCL11B*^+/+^ MEFs that had not been treated for osteoblast differentiation are represented as 1. All data are expressed as means ± S.E. (n = 3). (**b**) Cells at day 15 stained with Alizarin red.

Finally, we examined the role of *BCL11B* on osteoblastogenesis by culturing *BCL11B*^+/+^ and *BCL11B*^−/−^ MEFs for 15 days in the osteoblast differentiation medium. *BCL11B*^−/−^ MEFs differentiated into osteoblasts at comparable levels to *BCL11B*^+/+^ MEFs, based on the expression levels of the osteoblast markers *Runx2, Dlx5, Osteopontin*, and *Osterix,* and alizarin red staining (Fig. 7a,b). In contrast to adipogenesis, the mRNA expression of *BCL11B* did not increase during osteoblastogenesis, but instead slightly decreased between 0.5 and 7 d after differentiation (Fig. 7c).

## Discussion

In this study, we demonstrated that *BCL11B*^−/−^ mice have reduced subcutaneous WAT (Fig. 1b), and that 3T3-L1 cells in which *BCL11B* expression has been reduced by shRNA and MEFs from *BCL11B*^−/−^ embryos have a reduced ability to undergo adipogenesis (Figs 3 and 4). Furthermore, a DNA microarray analysis using samples from MEFs at 12 h after adipocyte differentiation indicated that *BCL11B*^−/−^ MEFs had an increased expression of *Wisp2* and *Osteopontin*, which are downstream genes of the Wnt/β-catenin signaling pathway, compared with *BCL11B*^+/+^ MEFs (Fig. 6a), suggesting that BCL11B suppresses this pathway. There are several possible explanations for this suppression. First, a loss in the *BCL11B* gene may stimulate the transcriptional activity of Tcf-β-catenin during adipocyte differentiation. We have previously reported that the introduction of BCL11B in human cell lines downregulates the transcription of Tcf-β-catenin target genes, and that the impairment of BCL11B promotes tumor development in mouse and human intestines, at least in part, through the deregulation of the β-catenin pathway^26^. It has also been reported that BCL11B is a mammalian SWI/SNF subunit^27^, another subunit of which (BRG1) interacts with β-catenin to stimulate the transcriptional activity of Tcf-β-catenin^28^. Second, BCL11B may suppress the Wnt/β-catenin signaling pathway by modulating the activity of COUP-TFII. COUP-TFII is a suppressive transcription factor, the expression of which has been reported to increase transiently during adipogenesis, in a similar way to *BCL11B*^29^. COUP-TFII suppresses *Wnt10b* gene expression by binding to the *Wnt10b* gene promoter, which inhibits the Wnt10b-mediated activation of the Wnt/β-catenin signaling pathway and thus stimulates adipogenesis^29^. It is considered that BCL11B, which is also known as COUP-TF-interacting protein 2 (CTIP2), functions as a co-repressor of COUP-TFs^9^. Thus, it is probable that a loss in the *BCL11B* gene attenuates the COUP-TFII-mediated suppression of *Wnt10b* gene expression, thereby activating the Wnt/β-catenin signaling pathway. Clearly, further studies are required to determine the molecular mechanism by which BCL11B suppresses the Wnt/β-catenin signaling pathway and whether this suppression contributes to the BCL11B-mediated stimulation of adipogenesis.

We observed that BCL11B expression transiently increased during adipogenesis, and that dexamethasone was responsible for inducing this (Fig. 2). It has previously been reported that expression of the adipogenic master regulators C/EBPβ and C/EBPδ transiently increases during differentiation, as does the expression of KLF4, with IBMX being responsible for inducing the latter^30^. At present, the molecular mechanism that is involved in the transient induction of BCL11B expression during adipogenesis is unclear. Dexamethasone is a type of synthetic glucocorticoid, and therefore, the glucocorticoid receptor is a candidate for stimulating *BCL11B* gene expression via dexamethasone. However, the *BCL11B* gene promoter (3 kb upstream from the transcription start site) was not found to contain a glucocorticoid receptor-responsive element; therefore, we cannot exclude the possibility that other transcription factors are involved in this regulation. Further studies are needed to determine the molecular mechanism of the transient induction of BCL11B expression during adipogenesis. It has been found that the treatment of D1 mesenchymal progenitor cells with dexamethasone stimulates their differentiation into adipocytes through suppression of the Wnt/β-catenin signaling pathway^8^. Thus, it is conceivable that acceleration of BCL11B expression is responsible for the dexamethasone-mediated suppression of the Wnt/β-catenin signaling pathway during adipogenesis.

We clearly demonstrated that a loss in *BCL11B* suppressed adipogenesis. However, unexpectedly, we found that the enforced expression of BCL11B also suppressed adipogenesis in 3T3-L1 cells (Fig. 5b). Given that BCL11B inhibited and stimulated the activity of PPARγ and C/EBPβ, respectively, and BCL11B expression transiently increased during adipogenesis, the constitutive expression of BCL11B may suppress adipogenesis by disturbing the activities of adipogenic master regulators such as PPARγ and C/EBPβ. These results also indicate that the transient induction of BCL11B expression is essential for proper adipogenesis. By contrast, we found that the ability of MEFs to undergo osteoblastogenesis was unaffected by a loss in the *BCL11B* gene (Fig. 7a,b) and that the expression of the *BCL11B* gene did not increase during osteoblastogenesis (Fig. 7c), suggesting that BCL11B expression in MEFs may not be sufficient to affect the Wnt/β-catenin signaling pathway during osteoblastogenesis.

Obesity is a risk factor for metabolic diseases such as diabetes, dyslipidemia, and atherosclerosis. Because obesity associated with the accumulation of intraabdominal (visceral) fat has a much greater risk of metabolic disease than obesity associated with subcutaneous (peripheral) fat^31^, it is considered that visceral and peripheral adipose tissues exhibit different physiological functions. A comprehensive analysis that investigated which developmental genes were differentially expressed in epididymal (visceral) and subcutaneous WAT of mice revealed that multiple genes, including *TBX15*, are differentially expressed in an adipose tissue depot-specific manner^32^. In the present study, we found that BCL11B was more highly expressed in the subcutaneous WAT than in the epididymal WAT, indicating that *BCL11B* may also be responsible for the difference in the physiological functions of intra-abdominal and subcutaneous WAT.

In conclusion, our study indicates that the *BCL11B* gene is required for proper adipogenesis, but not osteoblastogenesis, in 3T3-L1 cells and MEFs, and that the activation of C/EBPβ and the suppression of the Wnt/β-catenin signaling pathway by BCL11B is, at least in part, responsible for this regulation. We were unable to investigate the influence of the loss in the *BCL11B* gene in adult mice because *BCL11B*^−/−^ mice died within 24 h. However, we should be able to elucidate the function of BCL11B in living mice in the future by using mouse models with adipose tissue-specific loss or gain of BCL11B.

## Methods

### Materials

Insulin, dexamethasone, troglitazone, pioglitazone, Oil Red O, ascorbic acid, β-glycerophosphate, bone morphogenetic protein 2 (BMP2), and Alizarin Red S were purchased from Sigma; and 3-Isobutyl-1-methylxanthine (IBMX) was purchased from Wako.

### Antibodies

Monoclonal anti-FLAG (M2), anti-c-Myc (9E10), and anti-β-actin (AC-15) antibodies were purchased from Sigma; monoclonal anti-C/EBPβ (1H7) and rat monoclonal anti-BCL11B (25B6) antibodies were purchased from Abcam; polyclonal anti-BCL11B (NB600-262) antibody was purchased from Novus; and polyclonal anti-Perilipin (GP29) antibody was purchased from Progen.

### Media

Medium A contained Dulbecco’s Modified Eagle Medium (DMEM) supplemented with 100 units/mL penicillin, 100 μg/mL streptomycin, and 10% (v/v) calf serum. Medium B contained DMEM supplemented with 100 units/mL penicillin, 100 μg/mL streptomycin, and 10% (v/v) fetal bovine serum. The adipocyte differentiation medium was medium B supplemented with 0.5 mM IBMX, 1 μM dexamethasone, 100 μM ascorbic acid, and 10 μg/mL insulin. The adipocyte-growing medium was medium B supplemented with 100 μM ascorbic acid and 5 μg/mL insulin. The osteogenic medium was medium B supplemented with 100 μg/mL ascorbic acid, 10 mM β-glycerophosphate, and 50 ng/mL BMP2.

### Preparation of MEFs

Primary MEFs from embryos at embryonic day (E) 12.5 were isolated as previously described^33^. Then, differentiation experiments were performed using passage 2 cells.

### Cell culture

3T3-L1 preadipocytes and MEFs were cultured and differentiated into adipocytes as previously described^33^,^34^. Briefly, 3T3-L1 preadipocytes were grown to confluence in medium A. Two days after confluence, the cells were supplied with adipocyte differentiation medium, and 2 days after this, the cells were transferred to adipocyte growing medium and re-fed every 2 days. MEFs were differentiated using the adipocyte differentiation medium and 10 μM pioglitazone, and 3T3-L1 cells were differentiated using the adipocyte differentiation medium. MEFs were differentiated into osteoblasts as previously described^35^ with minor modifications. In brief, confluent MEFs were incubated in osteogenic medium and replaced every 2 days for up to 15 days.

### Plasmid constructs

Lentiviral plasmids were constructed for short hairpin RNA (shRNA) of mouse BCL11B or the control (Scramble II Duplex; Dharmacon) by recombining CS-RfA-EG (RIKEN) with pENTR4-H1 (RIKEN) inserted with oligonucleotide DNA for shRNA expression. The target sequences were as follows: BCL11B#1, 5′-GGGACAGCAATCCTTTCAACC-3′; BCL11B#2, 5′-GCTGCCAGGTGACCTGAAAGC-3′; and control, 5′-GCGCGCTTTGTAGGATTCG-3′. The expression plasmids for PPARγ, RXRα, and C/EBPs, and the luciferase reporter plasmids pPerilipin-Luc and p(C/EBP-RE) × 4-Luc have been described previously^34^,^36^,^37^. The expression plasmid for BCL11B-3 × FLAG was constructed by inserting fragments coding mouse BCL11B into p3 × FLAG-CMV14 (Sigma); and the expression retroviral plasmid for BCL11B-3 × FLAG was constructed by inserting fragments coding mouse BCL11B-3 × FLAG into pMXs-IRES-GFP^38^.

### Lentiviral and retroviral infection

Each virus and infected cells were produced following previously described methods^36^.

### Oil Red O and Alizarin Red S staining

The cells were fixed with 4% paraformaldehyde in phosphate buffered saline (PBS) for 1 h. Then, the cells were washed with PBS and stained with Oil Red O (0.5% Oil Red O in 2-propanol: ddw = 3:2 [v/v]) for 1 h or 1% Alizarin Red S for 15 min. Following this, the cells were washed twice with distilled water before photographing them.

### Real-time polymerase chain reaction (PCR)

Total RNA was extracted using Isogen (Nippon Gene), according to the manufacturer’s instructions. cDNA was synthesized and amplified from the total RNA using the High Capacity cDNA Reverse Transcription Kit (Applied Biosystems), and quantitative real-time PCR was performed using the Applied Biosystems 7000 Sequence Detection System. Relative mRNA levels were determined by normalizing the transcript levels to the 36B4 transcript levels. The TaqMan ID number and sequences of the primer sets are available upon request.

### Luciferase assays

3T3-L1 cells were plated in six-well plates at a density of 4 × 10^4^ cells, cultured with medium A for 24 h, and transfected with various plasmids, as indicated in the figure legends, using Lipofectamine^®^ 2000 (Invitrogen), according to the manufacturer’s instructions. Then, luciferase and β-galactosidase activities were determined 48 h later, as previously described^39^.

### Immunoprecipitation experiments

HEK293 cells were plated in six-well plates at a density of 4 × 10^5^ cells, cultured with medium B for 24 h, and transfected with various plasmids, as indicated in the figure legends, by the calcium phosphate method. The cells were lysed in RIPA buffer [50mM Tris-HCl (pH8.0), 150 mM NaCl, 0.1% SDS, 0.5% deoxycholate, and 1% TritonX-100] supplemented with protease inhibitors. After 2 days, the cells were lysed and immunoprecipitated with anti-FLAG antibodies and immunoblotted with antibodies indicated in the figure legends. 3T3-L1 cells were differentiated into adipocytes as described above. After 24 h, cells were lysed and immunoprecipitated with anti-BCL11B (NB600-262) antibodies and immunoblotted with antibodies indicated in the figure legends.

### DNA microarray analysis

Total RNA from *BCL11B*^+/+^ and *BCL11B*^−/−^ MEFs was extracted after 12 h of adipocyte differentiation using ISOGEN (Nippon Gene), according to the manufacturer’s instructions. Then, the total RNA was purified using an RNeasy^®^ mini kit (Qiagen), and its quality and quantity were confirmed. Following this, a DNA microarray analysis was performed as previously described^40^. Briefly, cDNA was synthesized from 100 ng of purified total RNA. Then, the cDNA was biotinylated and transcribed with T7 RNA polymerase. The purified aRNA was fragmented and hybridized to an Affymetrix Mouse Genome 430 2.0 Array (Affymetrix) at 45 °C for 16 h. Later, the array was washed and labeled with phycoerythrin. Fluorescence signals were scanned with the Affymetrix GeneChip System using Affymetrix GeneChip Command Console (AGCC) software to reduce the array images to the intensity of each probe (CEL files). The CEL files were quantified using the robust multi-array average (RMA)^41^ method with the statistical language R^42^ and Bioconductor^43^. The difference in gene expression between *BCL11B*^+/+^ and *BCL11B*^−/−^ MEFs was evaluated, and they were ranked using the Weighted Average Difference (WAD) gene ranking method^44^. Which genes ranked in the top 100 (50 increased genes and 50 decreased genes) was determined using TFacts program^45^ to estimate which transcription factors experienced changes in their activities in *BCL11B*^−/−^ MEFs. The data discussed in this publication have been deposited in NCBI’s Gene Expression Omnibus^46^ and are accessible through the GEO Series accession number GSE83980 (http://www.ncbi.nlm.nih.gov/geo/query/acc.cgi?acc=GSE83980).

### Mice and diet

All animal experiments were approved by the animal experiment committee of the University of Tokyo and performed in accordance with the relevant guidelines and regulations. The *BCL11B*^−/−^ mice of C57BL/6 background were generated, and genotyping of *BCL11B* was conducted as previously described^19^. The mice were housed in the animal care facility under controlled temperature and humidity conditions with a 12-h light/dark cycle. The mice were given free access to water and were fed a standard pelleted diet (Labo MR Stock; Nosan Corporation Bio Department).

### Morphological procedures

To observe the surface of the interscapular adipose tissue, newborn mice were anesthetized on ice cubes and sacrificed by decapitation. The dorsal skin was then removed from the intercostal area and the interscapular adipose tissue was observed with a binocular microscope. For Sudan III staining, embryos at E19.5 were sacrificed by decapitation, the viscera were removed, and then the remaining whole body was immersed in 10% buffered formaldehyde. After several days, each mouse open was cut, the chest was raised, and it was immersed in 30% sucrose overnight in a refrigerator. Then, the tissues were sectioned into 10-μm cryosections and stained with Sudan III.

### Statistical analysis

All data are presented as means ± S.E. Statistical analysis was performed using Ekuseru-Toukei Ver.2.0 (Social Survey Research Information). Comparisons between two treatments were made using Student’s *t-test*, whereas more than two treatments were compared using one-way analysis of variance (ANOVA) followed by the Bonferroni procedure. Differences were considered significant at *P* < 0.05.

## Additional Information

**How to cite this article**: Inoue, J. *et al*. Identification of BCL11B as a regulator of adipogenesis. *Sci. Rep.* **6**, 32750; doi: 10.1038/srep32750 (2016).
